# Di­chlorido­(4-{[(quinolin-2-yl)methyl­idene]amino}phenol-κ^2^ *N*,*N*′)mercury(II)

**DOI:** 10.1107/S1600536814007077

**Published:** 2014-04-09

**Authors:** Md. Serajul Haque Faizi, Pratik Sen

**Affiliations:** a Department of Chemistry, Indian Institute of Technology Kanpur, Kanpur, UP 208 016, India

## Abstract

In the mononuclear title complex, [HgCl_2_(C_16_H_12_N_2_O)], synthesized from the phenolic Schiff base 4-[(quinolin-2-yl­methyl­idene)amino]­phenol (QMAP), the coordination geometry around Hg^2+^ is distorted tetra­hedral, comprising two Cl atoms [Hg—Cl = 2.3565 (12) and 2.5219 (12) Å] and two N-atom donors from the QMAP ligand, *viz.* one imine and the other quinoline [Hg—N = 2.392 (2) and 2.237 (2) Å, respectively]. In the crystal, O—H⋯Cl hydrogen bonds generate a chain structure extending along the *c*-axis direction. Weak C—H⋯Cl and π–π stacking inter­actions [minimum ring centroid separation = 3.641 (3) Å] give an overall layered structure lying parallel to (001).

## Related literature   

For applications of 4-[(quinolin-2-ylmethylene)amino]phenol and related structures, see: Das *et al.* (2013 ▶); Jursic *et al.* (2002 ▶). For a related structure, see: Marjani *et al.* (2009 ▶).
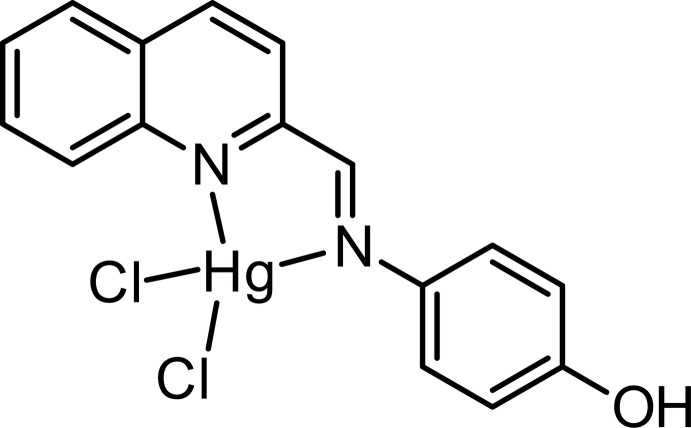


## Experimental   

### 

#### Crystal data  

[HgCl_2_(C_16_H_12_N_2_O)]
*M* *_r_* = 519.77
Monoclinic, 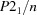

*a* = 7.539 (5) Å
*b* = 18.551 (5) Å
*c* = 10.806 (5) Å
β = 94.380 (5)°
*V* = 1506.9 (13) Å^3^
*Z* = 4
Mo *K*α radiation
μ = 10.57 mm^−1^
*T* = 100 K
0.29 × 0.19 × 0.12 mm

#### Data collection  

Bruker SMART APEX CCD diffractometer
Absorption correction: multi-scan (*SADABS*; Sheldrick, 2004 ▶) *T*_min_ = 0.143, *T*_max_ = 0.352
11156 measured reflections
2967 independent reflections
2679 reflections with *I* > 2σ(*I*)
*R*_int_ = 0.024

#### Refinement  

*R*[*F* ^2^ > 2σ(*F* ^2^)] = 0.016
*wR*(*F* ^2^) = 0.035
*S* = 1.05
2967 reflections
200 parameters
1 restraint
H-atom parameters constrained
Δρ_max_ = 0.65 e Å^−3^
Δρ_min_ = −0.40 e Å^−3^

### 

Data collection: *SMART* (Bruker, 2003 ▶); cell refinement: *SAINT* (Bruker, 2003 ▶); data reduction: *SAINT*; program(s) used to solve structure: *SIR97* (Altomare *et al.*, 1999 ▶); program(s) used to refine structure: *SHELXL97* (Sheldrick, 2008 ▶); molecular graphics: *DIAMOND* (Brandenberg & Putz, 2006 ▶); software used to prepare material for publication: *DIAMOND* (Brandenberg & Putz, 2006 ▶).

## Supplementary Material

Crystal structure: contains datablock(s) global, I. DOI: 10.1107/S1600536814007077/zs2293sup1.cif

Structure factors: contains datablock(s) I. DOI: 10.1107/S1600536814007077/zs2293Isup2.hkl

CCDC reference: 994407

Additional supporting information:  crystallographic information; 3D view; checkCIF report

## Figures and Tables

**Table 1:** Hydrogen-bond geometry (Å, °)

*D*—H⋯*A*	*D*—H	H⋯*A*	*D*⋯*A*	*D*—H⋯*A*
O1—H1⋯Cl2^i^	0.82	2.39	3.204 (3)	171
C7—H7⋯Cl2^ii^	0.92	2.78	3.644 (4)	156

Symmetry codes: (i) 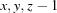; (ii) 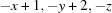.

**Fig. 1.**
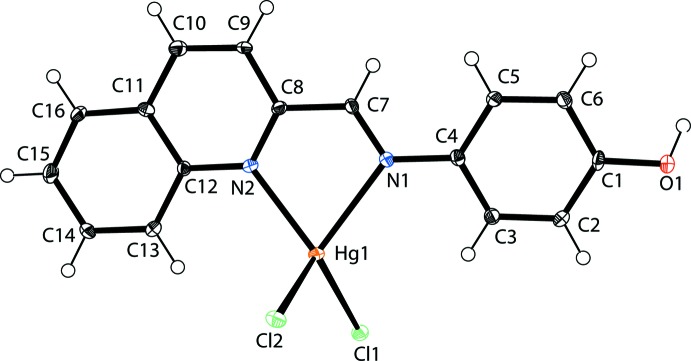
The molecular conformation and atom-numbering scheme for the title complex with non-H atoms drawn as 30% probability displacement ellipsoids.

**Fig. 2.**
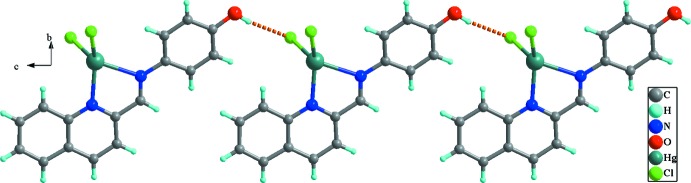
The one-dimensional hydrogen-bonded chain structure in the title complex extending along c, with hydrogen bonds shown as dashed lines.

**Fig. 3.**
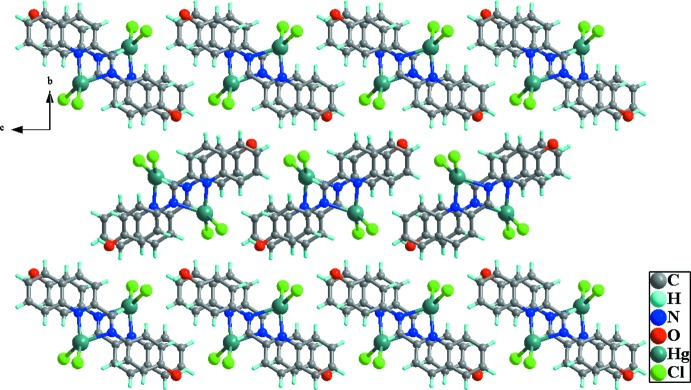
The two-dimensional structure viewed along the a-axial direction.

## supplementary crystallographic information

### 1. Comment

Quinoline derivatives of Schiff bases are important building blocks of many important compounds widely used in biological applications such as antioxidative and anticancer and fluorescent probe agents in industry and in coordination chemistry (Das *et al.*, 2013; Jursic *et al.*, 2002). The synthesis of polymeric complex of mercury(II) using the quinoline aldehyde derivative of the Schiff base 4-(quinolin-2-ylmethylene)aminophenol (QMAP) has not previously been reported. The title Hg^II^ complex with QMAP, [Hg(C_16_H_12_N_2_O)Cl_2_] has been synthesized and the structure is reported herein.

In the title mononuclear complex (Fig. 1) the HgCl_2_N_2_ coordination geometry is distorted tetrahedral, comprising two Cl-atoms [Hg1—Cl1 and Hg1—Cl2 = 2.3565 (12) and 2.5219 (12) Å respectively] and two N-atom donors from the QMAP ligand, one imine [Hg1–N1 = 2.392 (2) Å] and the other quinoline [Hg1—N2 = 2.237 (2) Å]. The observed Hg—Cl and Hg—N bond lengths and bond angles are considered normal for this type of Hg^II^ complex, *e.g.*, [Hg—N = 2.396 (4) Å] and [Hg—Cl = 2.367 (4) Å] (Marjani *et al.*, 2009). In the crystal, O1—H···Cl2 hydrogen bonds (Table 2) give a one-dimensional chain structure which extends along *c* (Fig. 2) and weak C7—H···Cl2 hydrogen bonds and π–π ring stacking interactions [minimum ring centroid separation between the inversion related benzene and quinoline rings = 3.641 (3) Å] give an overall two-dimensional layered structure lying parallel to (001) (Fig. 3).

### 2. Experimental

A mixture of 4-(quinolin-2-ylmethylene)aminophenol (QMAP) (0.10 g, 0.40 mmol), mercury(II) chloride (0.11 g, 0.40 mmol) and ethanol (5 ml) were stirred vigorously for 30 min, after which the precipitate was filtered off and dissolved in dimethylformamide. Crystals of the title complex suitable for X-ray analysis was obtained within 2 days by slow evaporation of the DMF solvent.

### 3. Refinement

All H-atoms were positioned geometrically and refined using a riding model with C—H = 0.92–0.93 Å and *U*_iso_(H) = 1.2*U*_eq_(C). The phenolic H-atom as located from a difference-Fourier map was also allowed to ride, with O—H = 0.83 (2) Å and, *U*_iso_(H) = 1.5*U*_eq_(O).

### Crystal data

**Table:**

[HgCl_2_(C_16_H_12_N_2_O)]	*F*(000) = 976
*M**_r_* = 519.77	*D*_x_ = 2.291 Mg m^−^^3^
Monoclinic, *P*2_1_/*n*	Mo *K*α radiation, λ = 0.71073 Å
Hall symbol: -P 2yn	Cell parameters from 999 reflections
*a* = 7.539 (5) Å	θ = 1.8–25.5°
*b* = 18.551 (5) Å	µ = 10.57 mm^−^^1^
*c* = 10.806 (5) Å	*T* = 100 K
β = 94.380 (5)°	Needle, yellow
*V* = 1506.9 (13) Å^3^	0.29 × 0.19 × 0.12 mm
*Z* = 4	

### Data collection

**Table:**

Bruker SMART APEX CCD diffractometer	2967 independent reflections
Radiation source: fine-focus sealed tube	2679 reflections with *I* > 2σ(*I*)
Graphite monochromator	*R*_int_ = 0.024
ω scans	θ_max_ = 26.0°, θ_min_ = 2.2°
Absorption correction: multi-scan (*SADABS*; Sheldrick, 2004)	*h* = −9→9
*T*_min_ = 0.143, *T*_max_ = 0.352	*k* = −22→22
11156 measured reflections	*l* = −13→10

### Refinement

**Table:**

Refinement on *F*^2^	Primary atom site location: structure-invariant direct methods
Least-squares matrix: full	Secondary atom site location: difference Fourier map
*R*[*F*^2^ > 2σ(*F*^2^)] = 0.016	Hydrogen site location: inferred from neighbouring sites
*wR*(*F*^2^) = 0.035	H-atom parameters constrained
*S* = 1.05	*w* = 1/[σ^2^(*F*_o_^2^) + (0.0146*P*)^2^ + 0.8024*P*] where *P* = (*F*_o_^2^ + 2*F*_c_^2^)/3
2967 reflections	(Δ/σ)_max_ < 0.001
200 parameters	Δρ_max_ = 0.65 e Å^−^^3^
1 restraint	Δρ_min_ = −0.40 e Å^−^^3^

### Special details

**Table:**

Geometry. All e.s.d.'s (except the e.s.d. in the dihedral angle between two l.s. planes) are estimated using the full covariance matrix. The cell e.s.d.'s are taken into account individually in the estimation of e.s.d.'s in distances, angles and torsion angles; correlations between e.s.d.'s in cell parameters are only used when they are defined by crystal symmetry. An approximate (isotropic) treatment of cell e.s.d.'s is used for estimating e.s.d.'s involving l.s. planes.
Refinement. Refinement of *F*^2^ against ALL reflections. The weighted *R*-factor *wR* and goodness of fit *S* are based on *F*^2^, conventional *R*-factors *R* are based on *F*, with *F* set to zero for negative *F*^2^. The threshold expression of *F*^2^ > σ(*F*^2^) is used only for calculating *R*-factors(gt) *etc*. and is not relevant to the choice of reflections for refinement. *R*-factors based on *F*^2^ are statistically about twice as large as those based on *F*, and *R*- factors based on ALL data will be even larger.

### Fractional atomic coordinates and isotropic or equivalent isotropic displacement parameters (Å^2^)

**Table:**

	*x*	*y*	*z*	*U*_iso_*/*U*_eq_	
C1	0.7674 (4)	1.15360 (15)	−0.3705 (3)	0.0140 (6)	
C2	0.8429 (4)	1.17912 (16)	−0.2579 (3)	0.0147 (6)	
H2	0.8987	1.2238	−0.2535	0.018*	
C3	0.8345 (4)	1.13750 (15)	−0.1520 (3)	0.0143 (6)	
H3	0.8842	1.1548	−0.0764	0.017*	
C4	0.7528 (4)	1.07023 (15)	−0.1574 (3)	0.0124 (6)	
C5	0.6783 (4)	1.04481 (16)	−0.2710 (3)	0.0141 (6)	
H5	0.6235	0.9999	−0.2757	0.017*	
C6	0.6857 (4)	1.08617 (15)	−0.3766 (3)	0.0140 (6)	
H6	0.6359	1.0689	−0.4522	0.017*	
C7	0.6910 (4)	0.96599 (16)	−0.0438 (3)	0.0124 (6)	
C8	0.6931 (4)	0.92330 (15)	0.0707 (3)	0.0120 (6)	
C9	0.6343 (4)	0.85181 (15)	0.0639 (3)	0.0135 (6)	
H9	0.5937	0.8318	−0.0119	0.016*	
C10	0.6374 (4)	0.81183 (16)	0.1701 (3)	0.0150 (6)	
H10	0.5981	0.7643	0.1671	0.018*	
C11	0.6999 (4)	0.84260 (15)	0.2837 (3)	0.0132 (6)	
C12	0.7564 (4)	0.91591 (15)	0.2848 (3)	0.0116 (6)	
C13	0.8157 (4)	0.94846 (16)	0.3981 (3)	0.0153 (6)	
H13	0.8537	0.9962	0.3994	0.018*	
C14	0.8176 (4)	0.91016 (16)	0.5057 (3)	0.0176 (7)	
H14	0.8556	0.9323	0.5803	0.021*	
C15	0.7628 (4)	0.83733 (16)	0.5061 (3)	0.0180 (7)	
H15	0.7656	0.8119	0.5804	0.022*	
C16	0.7061 (4)	0.80451 (16)	0.3977 (3)	0.0163 (6)	
H16	0.6709	0.7565	0.3984	0.020*	
N1	0.7487 (3)	1.03052 (12)	−0.0449 (2)	0.0113 (5)	
N2	0.7526 (3)	0.95410 (13)	0.1765 (2)	0.0117 (5)	
O1	0.7768 (3)	1.19596 (11)	−0.47207 (19)	0.0199 (5)	
H1	0.7324	1.1748	−0.5334	0.030*	
Hg1	0.832221 (15)	1.069766 (6)	0.162214 (10)	0.01536 (4)	
Cl1	1.07921 (9)	1.14792 (4)	0.18122 (6)	0.01560 (15)	
Cl2	0.59358 (10)	1.13188 (4)	0.27415 (7)	0.01732 (15)	
H7	0.6500	0.9430	−0.1160	0.0140*	

### Atomic displacement parameters (Å^2^)

**Table:**

	*U*^11^	*U*^22^	*U*^33^	*U*^12^	*U*^13^	*U*^23^
C1	0.0159 (15)	0.0118 (15)	0.0148 (14)	0.0042 (12)	0.0041 (12)	0.0050 (12)
C2	0.0147 (15)	0.0108 (15)	0.0188 (16)	0.0003 (12)	0.0022 (12)	0.0008 (12)
C3	0.0151 (15)	0.0153 (15)	0.0127 (14)	0.0015 (12)	0.0023 (12)	−0.0007 (12)
C4	0.0112 (14)	0.0138 (15)	0.0123 (14)	0.0043 (12)	0.0019 (11)	0.0004 (12)
C5	0.0157 (15)	0.0112 (14)	0.0154 (15)	−0.0003 (12)	0.0006 (12)	0.0001 (12)
C6	0.0163 (15)	0.0153 (16)	0.0103 (14)	0.0007 (12)	0.0008 (12)	−0.0011 (11)
C7	0.0122 (14)	0.0133 (15)	0.0116 (14)	0.0012 (12)	0.0011 (12)	−0.0029 (12)
C8	0.0099 (13)	0.0120 (15)	0.0142 (14)	0.0011 (12)	0.0011 (11)	−0.0008 (12)
C9	0.0154 (15)	0.0129 (15)	0.0122 (14)	−0.0018 (12)	0.0000 (12)	−0.0045 (12)
C10	0.0176 (15)	0.0090 (14)	0.0191 (15)	−0.0002 (12)	0.0052 (13)	−0.0016 (12)
C11	0.0132 (14)	0.0133 (15)	0.0134 (14)	0.0022 (12)	0.0034 (12)	−0.0010 (12)
C12	0.0100 (13)	0.0138 (15)	0.0114 (14)	0.0019 (11)	0.0033 (11)	0.0012 (11)
C13	0.0171 (15)	0.0106 (15)	0.0180 (15)	−0.0021 (12)	0.0009 (13)	−0.0001 (12)
C14	0.0237 (16)	0.0167 (16)	0.0121 (15)	−0.0006 (13)	−0.0014 (13)	−0.0023 (12)
C15	0.0221 (16)	0.0180 (16)	0.0135 (15)	0.0005 (13)	−0.0003 (13)	0.0046 (12)
C16	0.0211 (16)	0.0096 (15)	0.0183 (16)	−0.0006 (12)	0.0029 (13)	0.0019 (12)
N1	0.0116 (12)	0.0109 (13)	0.0114 (12)	0.0011 (10)	0.0018 (10)	−0.0005 (10)
N2	0.0120 (12)	0.0088 (12)	0.0143 (12)	0.0006 (10)	0.0008 (10)	0.0005 (10)
O1	0.0322 (13)	0.0140 (11)	0.0134 (10)	−0.0044 (10)	0.0012 (10)	0.0040 (9)
Hg1	0.02022 (7)	0.01239 (6)	0.01368 (6)	−0.00471 (5)	0.00259 (4)	−0.00206 (5)
Cl1	0.0152 (3)	0.0146 (4)	0.0169 (3)	−0.0022 (3)	0.0008 (3)	−0.0007 (3)
Cl2	0.0177 (4)	0.0182 (4)	0.0161 (4)	0.0002 (3)	0.0018 (3)	−0.0042 (3)

### Geometric parameters (Å, º)

**Table:**

C1—O1	1.356 (3)	C10—C11	1.402 (4)
C1—C2	1.386 (4)	C10—H10	0.9300
C1—C6	1.394 (4)	C11—C16	1.418 (4)
C2—C3	1.386 (4)	C11—C12	1.425 (4)
C2—H2	0.9300	C12—N2	1.366 (4)
C3—C4	1.391 (4)	C12—C13	1.407 (4)
C3—H3	0.9300	C13—C14	1.363 (4)
C4—C5	1.393 (4)	C13—H13	0.9300
C4—N1	1.424 (4)	C14—C15	1.413 (4)
C5—C6	1.379 (4)	C14—H14	0.9300
C5—H5	0.9300	C15—C16	1.360 (4)
C6—H6	0.9300	C15—H15	0.9300
C7—N1	1.274 (4)	C16—H16	0.9300
C7—C8	1.469 (4)	N1—Hg1	2.392 (2)
C7—H7	0.9170	N2—Hg1	2.237 (2)
C8—N2	1.325 (4)	O1—H1	0.8200
C8—C9	1.398 (4)	Hg1—Cl1	2.3565 (12)
C9—C10	1.365 (4)	Hg1—Cl2	2.5219 (12)
C9—H9	0.9300		
			
O1—C1—C2	118.0 (3)	C10—C11—C12	118.4 (3)
O1—C1—C6	122.2 (3)	C16—C11—C12	118.6 (3)
C2—C1—C6	119.9 (3)	N2—C12—C13	120.5 (3)
C3—C2—C1	119.5 (3)	N2—C12—C11	120.1 (3)
C3—C2—H2	120.2	C13—C12—C11	119.5 (3)
C1—C2—H2	120.2	C14—C13—C12	120.1 (3)
C2—C3—C4	120.9 (3)	C14—C13—H13	120.0
C2—C3—H3	119.6	C12—C13—H13	120.0
C4—C3—H3	119.6	C13—C14—C15	121.1 (3)
C3—C4—C5	119.2 (3)	C13—C14—H14	119.4
C3—C4—N1	117.8 (2)	C15—C14—H14	119.4
C5—C4—N1	122.9 (3)	C16—C15—C14	119.9 (3)
C6—C5—C4	120.1 (3)	C16—C15—H15	120.1
C6—C5—H5	119.9	C14—C15—H15	120.1
C4—C5—H5	119.9	C15—C16—C11	120.8 (3)
C5—C6—C1	120.4 (3)	C15—C16—H16	119.6
C5—C6—H6	119.8	C11—C16—H16	119.6
C1—C6—H6	119.8	C7—N1—C4	121.6 (2)
N1—C7—C8	122.2 (3)	C7—N1—Hg1	110.07 (19)
N1—C7—H7	121.0	C4—N1—Hg1	128.32 (18)
C8—C7—H7	116.0	C8—N2—C12	119.9 (2)
N2—C8—C9	122.6 (3)	C8—N2—Hg1	115.40 (19)
N2—C8—C7	118.4 (3)	C12—N2—Hg1	124.64 (19)
C9—C8—C7	119.0 (3)	C1—O1—H1	109.5
C10—C9—C8	119.1 (3)	N2—Hg1—Cl1	143.01 (6)
C10—C9—H9	120.4	N2—Hg1—N1	73.73 (8)
C8—C9—H9	120.5	Cl1—Hg1—N1	114.76 (6)
C9—C10—C11	119.8 (3)	N2—Hg1—Cl2	101.54 (7)
C9—C10—H10	120.1	Cl1—Hg1—Cl2	105.37 (4)
C11—C10—H10	120.1	N1—Hg1—Cl2	116.19 (6)
C10—C11—C16	123.0 (3)		
			
O1—C1—C2—C3	179.5 (3)	C12—C11—C16—C15	0.7 (4)
C6—C1—C2—C3	−0.7 (4)	C8—C7—N1—C4	177.3 (3)
C1—C2—C3—C4	0.6 (4)	C8—C7—N1—Hg1	−4.5 (3)
C2—C3—C4—C5	−0.2 (4)	C3—C4—N1—C7	−173.5 (3)
C2—C3—C4—N1	−179.8 (3)	C5—C4—N1—C7	6.8 (4)
C3—C4—C5—C6	−0.1 (4)	C3—C4—N1—Hg1	8.6 (4)
N1—C4—C5—C6	179.5 (3)	C5—C4—N1—Hg1	−171.1 (2)
C4—C5—C6—C1	−0.1 (4)	C9—C8—N2—C12	−0.6 (4)
O1—C1—C6—C5	−179.7 (3)	C7—C8—N2—C12	180.0 (2)
C2—C1—C6—C5	0.5 (4)	C9—C8—N2—Hg1	−178.6 (2)
N1—C7—C8—N2	2.0 (4)	C7—C8—N2—Hg1	2.1 (3)
N1—C7—C8—C9	−177.4 (3)	C13—C12—N2—C8	−178.7 (3)
N2—C8—C9—C10	0.2 (4)	C11—C12—N2—C8	1.3 (4)
C7—C8—C9—C10	179.5 (3)	C13—C12—N2—Hg1	−1.0 (4)
C8—C9—C10—C11	−0.3 (4)	C11—C12—N2—Hg1	178.98 (19)
C9—C10—C11—C16	179.7 (3)	C8—N2—Hg1—Cl1	−112.83 (19)
C9—C10—C11—C12	0.9 (4)	C12—N2—Hg1—Cl1	69.4 (2)
C10—C11—C12—N2	−1.4 (4)	C8—N2—Hg1—N1	−3.10 (19)
C16—C11—C12—N2	179.8 (3)	C12—N2—Hg1—N1	179.1 (2)
C10—C11—C12—C13	178.5 (3)	C8—N2—Hg1—Cl2	111.08 (19)
C16—C11—C12—C13	−0.3 (4)	C12—N2—Hg1—Cl2	−66.7 (2)
N2—C12—C13—C14	179.4 (3)	C7—N1—Hg1—N2	3.94 (18)
C11—C12—C13—C14	−0.5 (4)	C4—N1—Hg1—N2	−178.0 (2)
C12—C13—C14—C15	0.9 (5)	C7—N1—Hg1—Cl1	145.35 (17)
C13—C14—C15—C16	−0.4 (5)	C4—N1—Hg1—Cl1	−36.6 (2)
C14—C15—C16—C11	−0.4 (5)	C7—N1—Hg1—Cl2	−91.15 (19)
C10—C11—C16—C15	−178.0 (3)	C4—N1—Hg1—Cl2	87.0 (2)

### Hydrogen-bond geometry (Å, º)

**Table:**

*D*—H···*A*	*D*—H	H···*A*	*D*···*A*	*D*—H···*A*
O1—H1···Cl2^i^	0.82	2.39	3.204 (3)	171
C7—H7···Cl2^ii^	0.92	2.78	3.644 (4)	156

Symmetry codes: (i) *x*, *y*, *z*−1; (ii) −*x*+1, −*y*+2, −*z*.
